# TEMBLOR – Perspectives of EBI Database Services

**DOI:** 10.1002/cfg.133

**Published:** 2002-02

**Authors:** Henning Hermjakob, Rolf Apweiler

**Affiliations:** European Bioinformatics Institute Wellcome Trust Genome Campus Hinxton, Cambrige CB10 1SD UK


**Comparative and Functional Genomics**
*Comp Funct Genom* 2002; **3**: 47–50.
**DOI:** 10.1002/cfg.133

**Conference Review**

### TEMBLOR – Perspectives of EBI database services

A presentation for the ESF workshop 'Data integration in functional genomics and proteomics'

Henning Hermjakob* and Rolf Apweiler
*European Bioinformatics Institute, Wellcome Trust Genome Campus, Hinxton, Cambrige CB10 1SD, UK*

**Correspondence to:
European Bioinformatics Institute,
Wellcome Trust Genome
Campus, Hinxton, Cambrige
CB10 1SD, UK.
E-mail: hhe@ebi.ac.uk*

Received: 21 November 2001
Accepted: 27 November 2001
Published online:
13 December 2001

**Keywords: data integration; genomics; proteomics; protein–protein interaction**

Rapid progress in sequencing during the last few years has led to the publication of the sequences of around 70 complete genomes. At least twice that number is expected to be completed by the end of 2002. The availability of complete genomic data allows a whole range of new large-scale experiments such as the generation of whole-genome gene expression data, high-throughput protein identification by mass spectrometry, analysis of protein–protein interactions by two-hybrid-systems, phage-display, tandem–affinity–purification or other methods. These and other types of high-throughput experiments all produce large quantities of data, which must be stored in robust databases to enable their analysis and exploitation. Much of this data is currently spread over many databases with differing structures and locations making it difficult for users to have an integrated view of the information. To cope with data of such magnitude and complexity, higher interoperability of databases is essential.

Traditionally data distribution in the life science domain takes the form of exchanges of 'flat–files', ie., ASCII text files in a database-specific format. Commercial and academic data providers and end-users retrieve these data sets, write tools to parse them and reformat them usually in their own format to access them with their internal analysis tools. Due to the dramatic increase of the quantity and complexity of biological data, it is clear that distribution and storage of data in flat-files will have to be replaced in the future by more appropriate systems. Various initiatives in the domain, e.g. SRS [4] or Entrez [3], are focused on access to internal resources, by concentrating all the data in one central site and thus offering integrated views. The biggest drawback of such an approach is that these resources can only offer up-to-date data for data collections maintained on site. Data from external providers integrated in such a system will never be up-to-date, and updating and maintaining local copies of external data collections in such centralised databases or data warehouses is a major task.

Another approach is the federation of different databases; each located at a different centre. With all major molecular biology databases available on the web, this has happened, on a very low level, by the use of database cross-references providing links from one database to one or many other related resources. This linking is initially easy to achieve,

Copyright © 2001 John Wiley & Sons, Ltd.

will usually point users to the most recent data in a cross-referenced database, and makes maintenance and updating of local copies of external databases unnecessary. The drawback here is that this approach allows only linking on the coarse database entry level, and does not allow the computation across databases or integrated views. However, this may be still the method of choice to integrate small, specialised databases to core resources.

The InterPro [1] project, which provides an integrated view of the protein domain and family signature databases PROSITE, PRINTS, Pfam, SMART, TIGRFAM, and ProDom, as well as of the underlying protein sequence database SWISS-PROT + TrEMBL, uses a third way. InterPro tries to combine the advantages of a central database with the federated approach by creating a central integrative layer to store only the core data from its member databases and linking back to the richer data at the individual member databases. This approach allows minimising the hassle of updating and maintaining local copies of external data collections, while still allowing the computation across databases or integrated views. The same approach of building an integrative layer to unify resources while linking out to the original data source for more detailed information will be used in an initiative to create a next generation European bioinformatics resource.

A consortium of 25 leading scientific organisations from Europe and Israel, coordinated by the European Bioinformatics Institute, has initiated the TEMBLOR project, which is funded by the European Union and started in January 2002. The TEMBLOR project will provide a highly integrated view of genomic and proteomic data (Integr8) by drawing on databases maintained at major bioinformatics centres in Europe (Table 1). New resources for patent, protein–protein interaction (IntAct), structural (EMSD), and microarray (DESPRAD) data will be created or will move from prototype to production status (Figure 1). The Integr8 component will enable text-, structure- and sequence-based searches against a gene-centric view of all completed genomes. Zooming in on the sequence data linked to the gene will allow the user to see genomic, transcriptional, and protein sequences linked together. Each level will give direct access to the whole body of scientific knowledge about a given gene, transcript or protein. Evidence tags will allow users to trace the original source of data and to quickly distinguish eg., between experimentally verified and predicted data.

Building on the integrated database interface,

Table 1. Databases participating in the integration layer

| Resource | URL |
|---|---|
| ArrayExpress | http://www.ebi.ac.uk/arrayexpress/ |
| EMBL Nucleotide Sequence Database | http://www.ebi.ac.uk/embl/index.html |
| CATH | http://www.biochem.ucl.ac.uk/bsm/cath/ |
| DbSNP | http://www.ncbi.nlm.nih.gov/SNP/ |
| EnsEMBL | http://www.ensembl.org/ |
| Eukaryotic Promoter Database (EPD) and EPDEX | http://www.epd.isb-sib.ch/ |
| Families of Structurally Similar Proteins (FSSP) | http://www.ebi.ac.uk/dali/fssp/ |
| Gene Ontology (GO) | http://www.geneontology.org/ |
| Homology derived Secondary Structure of Proteins (HSSP) | http://www.sander.ebi.ac.uk/hssp/ |
| HOBACGEN | http://pbil.univ-lyon1.fr/databases/hobacgen.html |
| HOVERGEN | http://pbil.univ-lyon1.fr/databases/hovergen.html |
| Human Genic Bi-Allelic Sequences Database (HGBASE) | http://hgbase.cgr.ki.se/ |
| InterPro (includes PROSITE, PRINTS, Pfam, ProDom and SMART) | http://www.ebi.ac.uk/interpro/ |
| Protein Data Bank (PDB) / European Macromolecular Structure Database (EMSD) | http://msd.ebi.ac.uk/ |
| Resource Center/Primary Database (RZPD) | http://www.rzpd.de/ |
| SWISS-2DPAGE | http://www.expasy.org/ch/ch2d/ |
| SWISS-MODEL Repository | http://www.expasy.org/ch/swissmod/SM_3DCrunch_Search.html |
| SWISS-PROT + TrEMBL | http://www.expasy.org/ch/sprot/<br>http://www.ebi.ac.uk/swissprot/ |
| Transcription Factor Database (TRANSFAC) | http://transfac.gbf.de/TRANSFAC/index.html<br>http://www.biobase.de |
| trEST and trGEN | http://hits.isb-sib.ch/ |

Copyright © 2001 John Wiley & Sons, Ltd. 

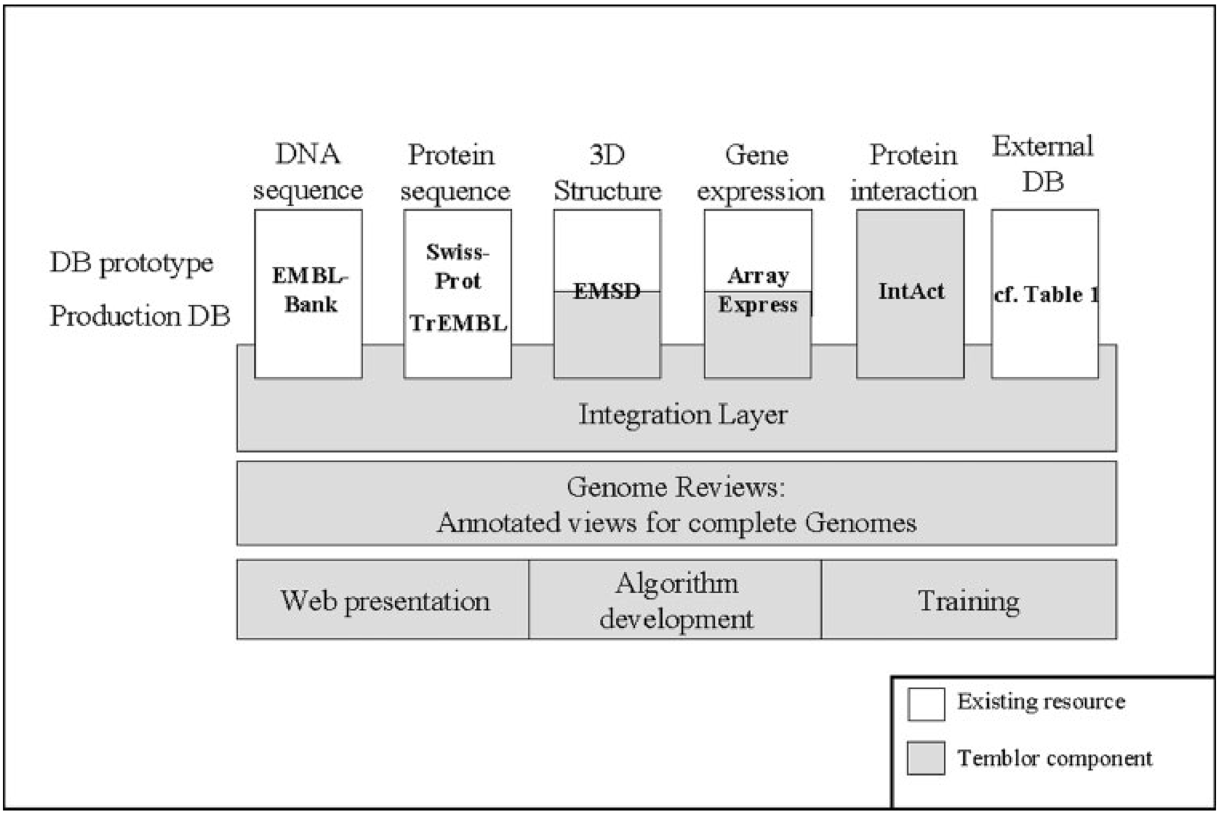

**Figure 1.** Temblor project structure new tools tools and algorithms will allow the user to perform complex analysis of the data, including whole genome/proteome comparison and complex strutural searches, eg., based on volume, surface and ligand chemistry. Queries will allow correlation of different data types, eg., expression data, protein–protein interaction data, and GO [5] annotation of the gene products involved. Application programming interfaces will be provided to allow complex third-party analysis of the data.

Specialised curators will maintain curated genome reviews and take up the 'proteomics challenge' through extensive crosslinking and data integration: While genomic data is relatively well accessible due to the requirements for deposition in public repositories, proteomics data is often highly fragmented across many species- or method-specific databases.

A central part of the TEMBLOR project are efforts for standardisation and international data exchange. In the framework of the *Microarray Gene Expression Database Group (MGED)* the *Microarray Gene Expression Markup Language (MAGE-ML)* and *Microarray Gene Expression Object Model (MAGE-OM)* standards for the description and exchange of microarray data will be further refined, and the ArrayExpress database will be developed as a public database for microarray data implementing these standards. The Macromolecular Structure Database at the EBI will further develop data exchange standards and tools for harvesting strutural data, and for data exchange with the Protein Data Bank (PDB). The IntAct project component will develop a standard for the representation and exchange of protein–protein interaction data and establish a public repository for protein–protein interaction data implementing this standard. A portable version of the repository software will allow easy in-house installation to facilitate the analysis of unpublished and confidential data in the context of publicly available data while at the same time allowing easy submission to the public repository after publication. In addition to third-party submissions, the IntAct protein–protein interaction database will be populated with experimental data from the project

Copyright © 2001 John Wiley & Sons, Ltd.

partners, and with manually curated reference data sets. As a long-term goal, the IntAct project will strive to establish an international data exchange for protein–protein interaction data, similar to the nucleotide sequence data exchange between EMBL, GenBank and DDBJ, to overcome the current fragmentation of publicly available protein–protein interaction data. Successful cooperative projects like GO, MGED, InterPro, or the EMBL/GenBank/DDBJ cooperation itself show the way to more collaboration, data integration and data exchange, which ultimately provides better resources to the entire scientific community in bioinformatics and molecular biology.

### References

1. Apweiler R, Attwood TK, Bairoch A, *et al.* 2001. The InterPro database, an integrated documentation resource for protein families, domans and functional sites. *Nucleic Acids Res* **29**: 37–40.
2. Berman HM, Westbrook J, Feng Z, *et al.* 2000. The Protein Data Bank. *Nucleic Acids Res* **28**: 235–242.
3. Entrez. **http://www.ncbi.nlm.nih.gov/Entrez/**
4. Etzold T, Ulyanov A, Argos P. 1996. SRS: information retrieval system for molecular biology data banks. *Meth Enzymol* **266**: 114–128.
5. The Gene Ontology Consortium. 2000. Gene Ontology: tool for the unification of biology. *Nat Genet* **25**: 25–29.

Copyright © 2001 John Wiley & Sons, Ltd.
